# Protein disorder prediction at multiple levels of sensitivity and specificity

**DOI:** 10.1186/1471-2164-9-S1-S9

**Published:** 2008-03-20

**Authors:** Joshua Hecker, Jack Y Yang, Jianlin Cheng

**Affiliations:** 1 School of Electrical Engineering and Computer Science, University of Central Florida, Orlando, FL 32816, USA; 2 Harvard University, PO Box 40088, Cambridge, Massachusetts 02140-0888, USA; 3 Computer Science Department and Informatics Institute, University of Missouri, Columbia, MO 65211, USA

## Abstract

**Background:**

Many protein regions and some entire proteins have no definite tertiary structure, existing instead as dynamic, disorder ensembles under different physiochemical circumstances. Identification of these protein disorder regions is important for protein production, protein structure prediction and determination, and protein function annotation. A number of different disorder prediction software and web services have been developed since the first predictor was designed by Dunker's lab in 1997. However, most of the software packages use a pre-defined threshold to select ordered or disordered residues. In many situations, users need to choose ordered or disordered residues at different sensitivity and specificity levels.

**Results:**

Here we benchmark a state of the art disorder predictor, DISpro, on a large protein disorder dataset created from Protein Data Bank and systematically evaluate the relationship of sensitivity and specificity. Also, we extend its functionality to allow users to trade off specificity and sensitivity by setting different decision thresholds. Moreover, we compare DISpro with seven other automated disorder predictors on the 95 protein targets used in the seventh edition of Critical Assessment of Techniques for Protein Structure Prediction (CASP7). DISpro is ranked as one of the best predictors.

**Conclusion:**

The evaluation and extension of DISpro make it a more valuable and useful tool for structural and functional genomics.

## Background

Prediction of protein structure from its sequence is one of most fundamental tasks of structural bioinformatics and proteomics. Although most amino acids (or residues) in most proteins adopt rather rigid structures (alpha-helix, beta-sheet, and loop), some residues in some proteins are very flexible and do not adopt a fixed conformation. The regions are usually called disorder regions [1]. Identification of protein disorder regions is important for protein production, protein function annotation, and protein structure prediction and determination [1]. For instance, flagging disorder residues is usually an important step in structural genomics projects.

To assist with the locating of these disordered regions, a number of computational tools have been developed which are capable of *predicting* the locations of the regions [2-12]. Most of these tools use a predefined threshold to choose ordered or disordered residues without allowing users to trade off the sensitivity and specificity, which is desirable in many different biological contexts. Moreover, a systematic benchmarking of the specificity-sensitivity relationship and the performance of different predictors, which provides a useful guide to better use these tools, is not available.

Thus, in this paper, we first create a large disorder dataset to evaluate the specificity-sensitivity relationship of a state of the art tool, DISpro [2]. We improve DISpro to allow users to set different thresholds to trade off the specificity and sensitivity of disorder predictions and to add a function for the visualization of protein disorder prediction.

Second, we benchmark several disorder predictors which participated in the seventh edition of Critical Assessment of Techniques for Protein Structure Prediction [13,14] on a common dataset. The evaluation provides a useful guide for the current state of the art of protein disorder prediction tools.

## Results

### Specificity and sensitivity at varying thresholds

To evaluate our modified DISpro, we utilized the 2408 sequence data set discussed in the second section. Each sequence was run through DISpro. For outputs, instead of using the basic DISpro cutoff of 0.5, 99 different threshold values from 0.01 to 0.99, in steps of 0.01, were used to select classification of ordered or disordered residues.

For analysis, we collected the total true positives (TP), false positives (FP), true negatives (TN), and false negatives (FN) for each threshold value, and calculated disordered residue prediction (SN) and ordered residue prediction (SP) values for each. The results are summarized in Table 1.

**Table 1:** Sensitivity and specificity over varying thresholds

Threshold	0.01	0.05	0.15	0.25	0.35	0.45	0.50	0.55	0.65	0.75	0.85	0.95	0.99
Sensitivity	0.98	0.85	0.72	0.64	0.57	0.51	0.49	0.46	0.39	0.32	0.24	0.13	0.01
Specificity	0.07	0.25	0.50	0.62	0.69	0.76	0.79	0.81	0.85	0.88	0.90	0.97	1.00

**Figure 1:**
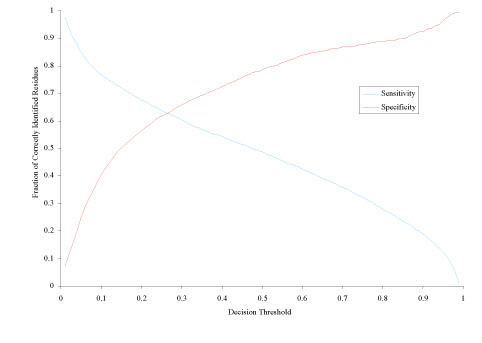
**Sensitivity and specificity over a varying decision threshold from 0.01 to 0.99, in steps of 0.01**.

The full set of output data can be better visualized in Figure 1, which contains plots of both SN and SP over a variable threshold value. Interestingly, both sensitivity and specificity lines follow somewhat S-shape slopes, with SN clearly decreasing as SP increasing.

**Figure 2:**
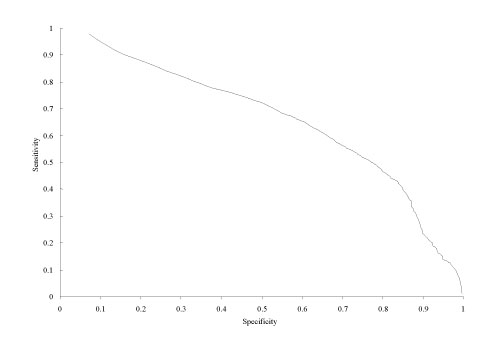
Sensitivity vs. specificity over varying threshold

To better illustrate the SN-SP relationship, we have included Figure 2, which presents these two performance criteria verse one another. This curve shows the direct connection between specificity and sensitivity, which is an inverse almost-linear relationship. As seen in Figures 1 and 2, our enhancement of DISpro to allow for variable threshold does in fact allow the user to modulate specificity and sensitivity values as they see fit. More so, we have demonstrated the control that a variable threshold gives to the user.

### Probability plot

**Figure 3:**
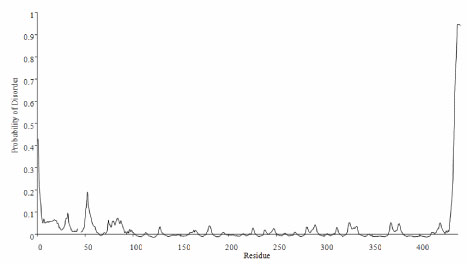
**Example output from modified DISpro. Displays probability of disorder for each residue in a sequence**.

Once testing of variable decision thresholds was completed, we finalized the DISpro add-on by incorporating user-friendly output from the program. Via Microsoft Excel, the user can view the statistical results from any run in graphical form, as seen in Figure 3. In this graph, users can visualize changes in likelihood of disorder from residue to residue across a test sequence. Ideally, output such as this would be employed by the user to select a specific decision threshold rather than that provided by the default in the original DISpro, thereby improving the range and capabilities of the software.

### Compare DISpro with seven disorder predictors on CASP7 dataset

DISpro and seven disorder predictors participated in the seventh edition of Critical Assessment of Techniques for Protein Structure Prediction (CASP7) [13,14]. CASP is the biannual community-wide evaluation of protein structure prediction techniques. CASP7 sent 100 protein targets whose structures were not yet known to the research groups around the world to predict their structures. The human and automated predictors from the research groups made predictions for these targets within a predefined period (two days for servers and a couple of weeks for humans) before the structures of the targets were released. The eight *automated* disorder predictors, including DISpro, DISOPRED [8], GeneSilico, MBI, BIME, DRIP-PRED [15], Distill [10], and ProfBval [16], participated in the disorder predictions of CASP7. Among them, GeneSilico is a meta-server using as inputs the outputs of other predictors and ProfBval was originally designed to predict the flexibility of residues in a protein sequence instead of disorder regions.

To benchmark these *automated* predictors, we downloaded their predictions for 95 official targets from the CASP7 web site (predictioncenter.org/casp7). We generate the states (order or disorder) for these targets using the structure files compiled by Dr. Yang Zhang (zhang.bioinformatics.ku.edu/casp7/native.html). We label residues without coordinates disordered and others ordered.

One caveat is that the dataset we created may be slightly different from the official disorder dataset used in CASP7 evaluation [17]. For instance, CASP7 uses at most 96 targets to evaluate disorder predictors, whereas we use only 95 official targets. However, the results based on the two datasets should be largely consistent. For instance, according to the official evaluation of CASP7, DISOPRED was ranked first and DISpro second in terms of ROC score (the area under the Receive Operator Curve). According to our evaluation on the dataset we created, both methods are also ranked among top two methods.

For the official CASP7 assessment of these predictors, readers should refer to the CASP7 disorder assessment paper [17] for details. Here we just try to provide a complementary evaluation of these predictors based on a common disorder definition.

**Figure 4:**
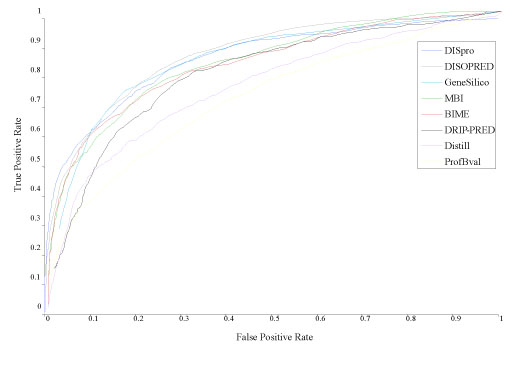
**ROC curves of eight predictors on the CASP7 dataset consisted of 95 protein targets**.

We compute the false positive rates (FP / (TN+FP)) and the true positive rates (TP / (TP + FN)) of the eight predictors for different decision thresholds. We plot the true positive rates against the false positive rates to generate ROC curves. Figure 4 shows the ROC curves of the eight predictors. We also compute the area (ROC score) of the ROC curve of each predictor to evaluate their performance. Table 2 reports the ROC scores of eight predictors. The results show that DISpro and DISOPRED are the top two predictors with ROC scores of 0.864 and 0.862, respectively.

**Table 2:** The ROC scores of eight predictors on the CASP7 dataset

Predictor	ROC score
DISpro	0.864
DISOPRED	0.862
GeneSilico	0.851
MBI	0.839
BIME	0.834
DRIP-PRED	0.804
Distill	0.757
ProfBval	0.710

It is worth noting that the accuracy of the automated disorder predictors is slightly lower than the best human predictors [9,11] in CASP7 [2]. However, a comparison with the human predictors cannot be made since their prediction data are not publicly available.

### Software usage

Installation and use of this add-on script follows the general protocol of the SCRATCH [18] protein data mining suite. Usage will require a pre-existing installation of SSpro [18,19], and the add-on program can either be added into the *script* folder of the DISpro package, or simply installed automatically with an updated version of DISpro.

Input to the program calls for a text file containing the test sequence in FASTA format, a file name to be used for data output, and a threshold value. The output file is intended to be used directly with Microsoft Excel to allow for quick and easy viewing of data trends in a graphical format. As with the rest of the SCRATCH suite, the DISpro add-on is designed for the Linux operation system, with all testing being done using Linux. The original DISpro package is available at: *contact.ics.uci.edu/download.html.* The add-on program is available at: babbage.cs.missouri.edu/~chengji/cheng_software.html.

## Method

### Data

The protein sequences used for testing were acquired from the Protein Data Bank (PDB) [20]. This dataset consisted of 3131 sequences, with a disorder residue frequency of 5.4% (54,364). As similarly noted [2], the majority of disordered regions were located at the N- and C- termini ends of the protein sequences.

To strictly evaluate the performance of DISpro, we removed all sequences previously used in the training or testing of the original DISpro. Thus, classification accuracy is based solely on data previously unseen by the network.

**Figure 5:**
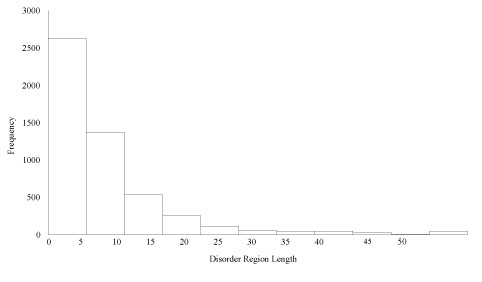
**Frequency of lengths of disordered regions**.

The remaining 2408 sequences were then analyzed for disorder residue frequency and region length. Out of a total 799,153 residues in the PDB dataset, 5.1% (40,455) of the residues were marked as disordered. Of those 40,455 disordered residues, 18.7% (7552) were located in regions of length greater than 30 amino acids. Overall frequency of region length can be seen in Figure 5.

### Input and output of neural network

The overall neural network system remains unchanged from the original DISpro, but it is discussed here briefly to ensure clarity. As in [2], DISpro utilizes a 1-dimensional recursive neural network, which we will refer to as 1D-RNN [2]. Please see Baldi and Pollastri (2003) for a detailed explanation of the 1D-RNN's rolling "wheel" system [21].

In the 1D-RNN architecture, the network is designed such that it can accept an entire sequence at once, rather than the more common sliding window technique, thereby allowing for variable input size. As an example, let us use a sequence of arbitrary length *I*. In this case, *I* represents the total number of residues in the example sequence, and I_*i*_ is a vector containing the 25 values used to represent residue *i*. Of these values, 20 represent the frequencies of the 20 amino acids from a PSI-BLAST profile [22], and the other five are binary values denoting secondary structure and solvent accessibility predictions [18,19,23].

For an output value, the 1D-RNN produce a vector of real numbers *O*, where *O*_*i*_ is the probability that residue i will be disordered. These probabilities are then utilized by DISpro to select a classification of disordered or ordered, based on a decision threshold of 0.5 [2]. However, by varying this threshold (as discussed in the next subsection), we are able to investigate the relationship of specificity and sensitivity of disorder predictions.

### Benchmarking sensitivity and specificity by varying the decision threshold

One key goal of this study is to investigate the specificity and sensitivity relationship of disorder prediction. The major difference between the original DISpro and our extended version is found at the final stage of data classification. While the original DISpro makes a classification decision based on the default threshold of 0.5, where ≤ 0.5 is ordered and >0.5 is disordered, we have now implemented the capability to vary the decision threshold as needed. As a result, users will be able to input their own threshold value and view the corresponding output.

To measure the effect of varying decision threshold, we compute the sensitivity and specificity of different decision thresholds. Sensitivity (TP / (TP + FN)) is the percentage of true disordered residues being predicted as disordered, while specificity (TP / (TP+FP)) is the percentage of predicted disordered regions being true disordered residues. TP, FP, FN and TN denote the number of true positives, false positives, false negatives, and true negatives.

### Benchmarking disorder predictors on CASP7 data

The other major goal of the study is to estimate the state of the art of the current disorder predictors. Thus, we systematically evaluate the performance of eight disorder region predictors on the CASP7 dataset including 95 proteins. We compute the true positive rates (TP / (TP + FN)) at different false positive rates (FP / (TN + FP)) to generate ROC curves of these predictors. We use the areas under the ROC curve (ROC scores) to compare these predictors.

## Conclusions and Future Work

In this study, we have created a large dataset to systematically investigate the performance of a state of the art protein disorder predictor, DISpro. We improve the predictor by allowing for variable threshold selection rather than a fixed default, and provide an easy transition from output data into graphical form. Our results demonstrate the effectiveness of a variable decision threshold, which sometime allows for a significant increase in the sensitivity with only a small drop in the specificity. Users can also visualize the predicted output probabilities of being disordered for all residues when making a decision on threshold values for their purpose.

Moreover, we benchmark DISpro with seven other protein disorder predictors on the 95 targets used in CASP7. The evaluation provides an approximate guide about the state of the art of the current protein disorder prediction methods.

In the future, we plan to use machine learning ensemble (or bagging) techniques to integrate different predictors evaluated in this research and some other predictors such as IUP [12] together to improve disorder prediction.

## Competing interests

The authors declare that they have no competing interests.

## Authors' contributions

JC conceived the project. JH and JC carried out experiments. JH and JC authored the first draft of manuscript. JY contributed to the biological significance and future work. JC, JH and JY edited the final manuscript and approved it.
